# Cardiac protein expression patterns are associated with distinct inborn exercise capacity in non-selectively bred rats

**DOI:** 10.1590/1414-431X20177033

**Published:** 2018-01-11

**Authors:** L.P. Ribeiro, L.C. Freitas-Lima, G.B. Naumann, S.S. Meyrelles, W. Lunz, S.F. Pires, H.M. Andrade, J.B.T. Carnielli, S.G. Figueiredo

**Affiliations:** 1 Departamento de Ciências Fisiológicas, Universidade Federal do Espírito Santo, Vitória, ES, Brasil; 2 Diretoria de Pesquisa e Desenvolvimento, Fundação Ezequiel Dias, Belo Horizonte, MG, Brasil; 3 Centro de Educação Física e Desportos, Universidade Federal do Espírito Santo, Vitória, ES, Brasil; 4 Departamento de Parasitologia, Universidade Federal de Minas Gerais, Belo Horizonte, MG, Brasil; 5 Núcleo de Doenças Infecciosas, Universidade Federal do Espírito Santo, Vitória, ES, Brasil

**Keywords:** Inborn aerobic capacity, Proteomics, 2-DE, MS, Heart proteins

## Abstract

In the present study, we successfully demonstrated for the first time the existence of cardiac proteomic differences between non-selectively bred rats with distinct intrinsic exercise capacities. A proteomic approach based on two-dimensional gel electrophoresis coupled to mass spectrometry was used to study the left ventricle (LV) tissue proteome of rats with distinct intrinsic exercise capacity. Low running performance (LRP) and high running performance (HRP) rats were categorized by a treadmill exercise test, according to distance run to exhaustion. The running capacity of HRPs was 3.5-fold greater than LRPs. Protein profiling revealed 29 differences between HRP and LRP rats (15 proteins were identified). We detected alterations in components involved in metabolism, antioxidant and stress response, microfibrillar and cytoskeletal proteins. Contractile proteins were upregulated in the LVs of HRP rats (α-myosin heavy chain-6, myosin light chain-1 and creatine kinase), whereas the LVs of LRP rats exhibited upregulation in proteins associated with stress response (aldehyde dehydrogenase 2, α-crystallin B chain and HSPβ-2). In addition, the cytoskeletal proteins desmin and α-actin were upregulated in LRPs. Taken together, our results suggest that the increased contractile protein levels in HRP rats partly accounted for their improved exercise capacity, and that proteins considered risk factors to the development of cardiovascular disease were expressed in higher amounts in LRP animals.

## Introduction

Aerobic capacity is a complex trait defined as the efficiency of using atmospheric oxygen as an electron acceptor in energy transfer processes. The primary limiting factor of aerobic capacity is the heart’s ability to maintain a sufficient cardiac output to support tissue energy demands. Lower levels of aerobic fitness have been associated with increased cardiovascular disease and mortality.

Heritability studies have demonstrated that 70 to 90% of the variation in aerobic capacity is genetically determined (1,2). Two genetic pathways contribute to an organism’s natural aerobic capacity phenotype: first, a set of genes determining variations in aerobic capacity in the untrained state (1,2), called intrinsic aerobic capacity; and second, another set of genes appearing to be responsible for the adaptive response to exercise (3). The aerobic capacity of an organism is therefore the sum of the expression of its intrinsic and adaptive genes in interaction with the environment (4). It is noteworthy that one important part of this heritability relies on cardiac muscle activity (5).

Specific rat genetic models of intrinsic low-capacity runners (LCRs) and high-capacity runners (HCRs) were developed by artificial selective breeding based on their ability to run on a treadmill (4,5). At generation 3, these lines differed in running capacity by 114% (5). After eleven generations, HCRs showed 347% higher aerobic capacity and better cardiac function when compared to LCRs (6). HCRs also presented higher stroke volume and cardiac output (5). At the cellular level, it has been demonstrated that HCRs show greater amplitude of calcium transients and higher efficiency in energy production (7), along with faster sarcomeric shortening and relaxation (6,8). In addition to low aerobic capacity, LCRs presented higher levels of fasting blood glucose, insulin, triglycerides and fatty acids, besides showing diminished vascular function compared to HCRs (6).

It has been shown through sequential mating that the two strains move towards extremes of organic functioning, including greater longevity in the HCR group (9). These data also indicate that there are clear metabolic and cardiac differences between rats with distinct intrinsic aerobic capacities resulting from divergent artificial selection. Additionally, differences in calcium transients and cardiomyocyte contraction, as well as differences in the expression of SERCA2a and the ryanodine receptor type 2, seem to emerge in rats selected - but not bred - by aerobic exercise capacity (10). More recently, changes in the levels of several cardiac tissue proteins were found between HCR and LCR rats from the second generation, which were associated with phenotype aerobic capacity (11).

Although the molecular bases involved in aerobic capacity phenotype are partly understood in selectively bred rats, the understanding of the process in non-selectively bred rats (intrinsic aerobic capacity) is lagging behind. Therefore, in this study we applied global proteomic profiling to investigate cardiac protein differences arising in non-selectively bred rats categorized by aerobic capacity.

## Material and Methods

### Animals

Male Fisher rats were obtained from the School of Nutrition animal housing facilities of the Universidade Federal de Ouro Preto, Ouro Preto, MG, Brazil, and housed (5 per cage) in a temperature-controlled environment (22-24°C) with a 12-h light/12-h dark cycle with access to water and rat chow *ad libitum*. All procedures of the present study were approved by the Ethics Committee of Universidade Federal do Espírito Santo (No. 079/2011).

### Exercise test protocol and animal selection

Selection for intrinsic exercise capacity (i.e., untrained rats) was conducted with minimum alterations according to protocols described by Prímola-Gomes et al. (10), which are based on distance run to exhaustion (DRE) on a motorized rodent treadmill (Insight, EP 131, Brazil).

Before exercise tests, the animals were familiarized with a motor-driven treadmill by running 5 min for 5 consecutive days (5° slope) at incremental speeds (10-15 m/min). The treadmill electric shock grid stimulus was set at 3 mA. This amount of exposure to treadmill running is below that required to produce a significant change in aerobic capacity (12). Animals that were unable to adapt to the treadmill, meaning that they were not even able to stand upright on it, were deemed unfit and excluded from the study (4).

During the first week, each rat was assessed for running capacity until exhaustion, once a day for 5 consecutive days. All test trials were performed at a starting belt velocity of 10 m/min at a constant slope of 5°. The belt speed velocity was then increased by 2 m/min every 2 min. Exhaustion was attained at the third time when the rat could no longer keep pace with the belt speed and remained on the shock grid for 2 s. Rats were selected according to their best running performance amongst five trials, which was considered the trial most closely associated with the heritable component of endurance running capacity. At the end of each trial, the rat was weighed and the respective DRE was registered.

A total of 73 male rats, aged 10 weeks and weighing ≅250 g, were assigned into two groups: low-running performance (LRP) and high-running performance (HRP). To be included in the LRP group, an animal had to have a DRE value less than one standard deviation below the population average. Otherwise, to be included in the HRP group, animals had to have a DRE value higher than one standard deviation above the population average.

### Tissue sampling

Left ventricles (LV) were harvested for proteomic analysis 2 weeks after the last day of exercise testing. Rats were weighed and killed by decapitation under resting conditions (30 mg/kg ketamine and 3 mg/kg xylazine). Their hearts were then isolated and washed with iced saline (three times) and iced Milli-Q water (twice) before being blotted dry and weighed. LVs were then removed, weighed, frozen in liquid nitrogen and kept at -80°C until use.

### Sample preparation

To proceed with the proteomic analysis, we selected LV tissue only from the 6 rats of each group that obtained the highest (HRP group) and lowest (LRP group) DRE values. The 6 samples of LV from each experimental group were processed together to create a pooled sample of each group. The pool obtained from each group was used for two-dimensional electrophoresis (2DE) comparative studies. LV protein extraction was performed as previously described (13) with some modifications. Briefly, LV portions were pulverized in liquid nitrogen and then a fraction (≅600 mg) of each powdered pool was homogenized by an ultrasonic sonicator (XL-2000, Q Sonica, USA) on ice in 10 volumes of 2D lysis buffer (8 M urea, 2 M thiourea, 4% CHAPS, 40 mM Tris base, and 0.2% protease inhibitor cocktail, Sigma-Aldrich, USA). Next, the homogenates were centrifuged (12,000 *g* for 45 min at 4°C) and the supernatants were collected and assayed for protein quantification (2D Quant Kit, GE Healthcare, USA). Aliquots of the protein extracts were split into single-use samples and stored at -80°C until use.

### Two-dimensional gel electrophoresis (2DE)

Protein samples (1000 μg of total soluble protein) were diluted in 350 µL Destreak rehydration solution (GE Healthcare), supplemented with 0.2% ampholytes 3-10, and applied to IPG strips (pH 3-10 NL -17 cm, Bio-Rad, USA) by in-gel rehydration. All isoelectric focusing (maximum 50 mA/strip) was performed on a Protean® IEF cell system (Bio-Rad) at 20°C. Electrical conditions were set as described by the supplier: active rehydration (50 V) for 12 h; step 1) 300 V constant for 3 h; step 2) gradient from 300 to 10,000 V over 2 h; step 3) 10,000 V until complete 60,000 Vh; step 4) 500 V constant for 5 h. Upon IEF completion, the IPG gel strip was incubated at room temperature for 15 min in equilibration buffer (50 mM Tris-HCl pH 8.8, 6 M urea, 2% SDS, 30% glycerol, and traces of bromophenol blue) containing 10 mg/mL DTT, followed by a second incubation step (15 min at room temperature) in equilibration buffer containing 25 mg/mL iodoacetamide instead of DTT.

The second dimension of electrophoresis was performed in a vertical system (Protean XL multi-cell Syste, Bio-Rad), with uniform 12% separating polyacrylamide gels at 15°C, initially at a constant current of 16 mA/gel for 30 min and then at 24 mA/gel until the dye front reached the bottom edge of the gel. Gels were stained with colloidal Coomassie Brilliant Blue G-250 according to Neuhoff et al. (14). All samples were run in technical triplicate. Gels were then scanned using an ImageScanner III calibrated densitometer (GE Healthcare).

### 2DE image analysis

Digitized 2DE gel images were analyzed using the Image Master 2D Platinum software (Version 7.05; GE Healthcare). Each sample was analyzed in technical triplicate. The authenticity and outline of each spot were validated by visual inspection and edited when necessary. The intensity of each protein spot was normalized by the total abundance of all valid spots. Spots that exhibited significant differences between the groups with different phenotypes (LRP and HRP) were selected for protein identification by mass spectrometry.

### Identification of spots by mass spectrometry (MS)

Protein spots with differential intensity were manually excised from the gel. Gel pieces were then destained by three 15-min rinses with 50% acetonitrile in 25 mM ammonium bicarbonate, followed by pure acetonitrile washing for 5 min, before being dried by SpeedVac (Eppendorf, USA). Dried gel fragments were then rehydrated with 10 μL of protease solution (Trypsin Gold, Mass Spectrometry Grade, Promega, USA, at 20 ng/μL in 50 mM ammonium bicarbonate) for 30 min on ice. Twenty microliters of 50 mM ammonium bicarbonate was then added and digestion was carried out for 16 h at 37°C. The peptides obtained by digestion were extracted from the gel by washing twice with 30 μL of 50% acetonitrile/5% formic acid with constant shaking for 30 min each. Trypsin digests were concentrated in a SpeedVac (Eppendorf) to approximately 10 μL. The protein digests were subjected to a desalting step using Zip-Tip (C18 resin; P10, Millipore, USA) as previously reported (15).

After that, the desalted tryptic peptide samples (1 μL) were co-crystallized with a matrix (0.25 µL of a saturated solution of α-cyano-4-hydroxycinnamic acid matrix (Aldrich, USA), in 70% acetonitrile/0.1% trifluoroacetic acid) onto an MTP AnchorChip™ 384 TF MALDI plate (BrukerDaltonics, Germany) and air-dried before loading into the MALDI ToF/ToF mass spectrometer. Mass spectra were acquired in positive reflector mode on a Bruker AutoflexIII™ MALDI ToF/ToF instrument (BrukerDaltonics). An external peptide calibration standard containing Bradykinin ([M+H]+ 757.39), Angiotensin II ([M+H]+ 1046.54), Angiotensin I ([M+H]+ 1296.68), Substance P ([M+H]+ 1347.74), Bombesin ([M+H]+1619.82), Renin substrate ([M+H]+ 1758.93), ACTH clip 1-17 ([M+H]+ 2093.09), ACTH clip 18-39 ([M+H]+ 2465.20) and Somatostatin 28 ([M+H]+ 3147.47) (BrukerDaltonics) was used to calibrate the instrument.

A list of peptides was obtained using the SNAP algorithm (Flexanalysis™, BrukerDaltonics). The most intense peaks were selected for MS/MS scan to obtain as many CID spectra as possible. MS and MS/MS spectra were combined by BioTools (BrukerDaltonics) and used to search against the NCBI non-redundant database using the Mascot® software (http://www.matrixscience.com). Search parameters were as follows: no restrictions on protein molecular weight; Rattus taxonomic; only tryptic peptides with one missed cleavage were considered; fixed and variable modifications were carbamidomethylation of Cys residues and Met-oxidation, respectively; mass accuracy of 0.8 Da was acceptable for matching peptides (MS and MS/MS mode).

Gel fragments with no protein from a bovine albumin molecular weight standard were used as negative and positive controls, respectively. Proteins were considered identified when homology with *Rattus norvegicus* species with a global Mascot score at α<0.05 was observed. Biological process categorization was based upon information provided by Gene Ontology (GO) and Panther (16) databases classification system (for *Rattus norvegicus*).

### Western blotting

Briefly, 50 μg of protein extract of LV from four individual animals of each group (HRP and LRP) were resolved on 12.5% SDS-PAGE and transferred onto nitrocellulose membranes (Hybond, UK), using a trans-blot wet transfer unit (Bio-Rad) by applying a current of 100 mA at 4°C overnight. Equal total protein loads were confirmed by Ponceau red (0.1% Ponceau red, 5% acetic acid, v/v) staining of the nitrocellulose membranes. The membranes were rinsed with TBS-Tween 0.1% and incubated with blocking buffer (5% low-fat milk powder in TBS-Tween 0.1%) at room temperature for 2 hours. Trans-blotted proteins were then probed overnight at 4°C with a rabbit polyclonal anti-desmin antibody (Santa Cruz, USA) at dilutions of 1:500. After washing three times in TBS-Tween 0.1% for 5 min, the membranes were incubated with horseradish peroxidase (HRP)-conjugated secondary antibody (1:5000 dilution) in blocking solution for 2 h.

Specific binding was revealed with a western blotting detection ECL system (Amersham, UK) and exposed to a CCD camera (ChemiDoc, Bio-Rad). Desmin signals were processed by Image Lab Software (Bio-Rad) and the signal intensities were measured in delimitated areas of equal size and reported as arbitrary units.

### Statistical analysis

The data (body weight, heart weight, heart weight-to-body weight ratio and intensity of protein spots and of desmin) are reported as means±SE. The differences between HRP and LRP groups were analyzed using an unpaired Student’s *t*-test (GraphPad Prism 5.03; GraphPad Software, USA) and were considered to be significant at P<0.05.

## Results

### Animal selection

The selection for intrinsic running capacity (aerobic exercise) was based upon the DRE on a motorized treadmill using a velocity-ramped running protocol. Ten of the 83 rats were unable to adapt to the treadmill and were excluded from the study, whereas the remaining 73 rats were subjected to aerobic endurance running capacity tests.

**Figure 1.**
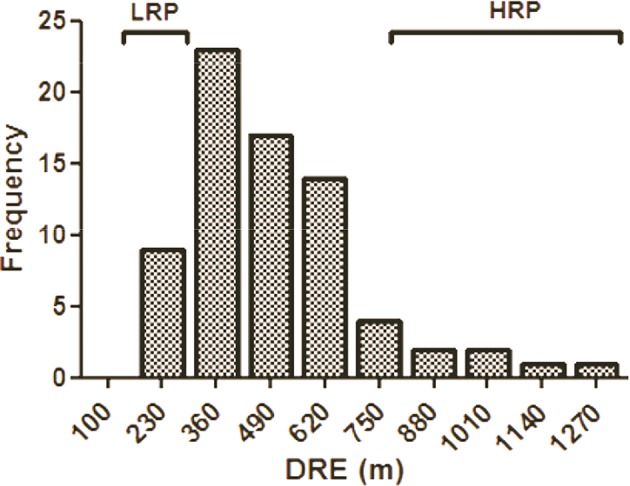
Frequency distribution of rats submitted to a progressive exercise test. The frequency is shown as total distance run to exhaustion (DRE; n=73 rats). LRP: Low running performance (9 rats); HRP: High running performance (9 rats).

Rats were selected as described above. The trial most closely associated with the heritable component of endurance running capacity was then used to construct a frequency histogram (Figure 1); the average DRE of the population was 504.0±24.7 m.

According to the adopted criterion (4), this protocol successfully categorized the groups: low running performance (LRP) and high running performance (HRP) (Figure 1). Rats that ran less than 293.0 m were included in the LRP group (12.3%, 9 animals) and rats that ran more than 715.2 m were included in the HRP group (12.3%, 9 animals). Rats with DRE values between 293.1 and 715.1 m (75.4%, 55 animals) were excluded from the study.

Next, we compared the parameters of HRP and LRP groups and assembled the results in Table 1. This analysis revealed that the running capacity of HRPs was approximately 3.5-fold higher than that of LRPs. Body weight, heart weight and the heart weight-to-body weight ratio were not significantly different between groups.

**Table 1.** Parameters of low running performance (LRP) and high running performance (HRP) groups.

Variable	LRP	HRP
Distance run to exhaustion (m)*	270.17±5.27	939.36±57.99§
Body weight (g)*	266.3±9.0	251.2±6.1
Heart weight (mg)	748.0±33.4	720.83±18.0
HW/BW (mg/g)	2.42±0.05	2.35±0.05

Data are reported as means±SE. Body weight: total body weight on its best performance in the maximal running test; HW/BW: heart weight-to-total body weight ratio of the animals when sacrificed (n=6 animals per group).

§ P<0.05 compared to LRP group (*t*-test).

* n=9 animals for LRP and HRP groups.

### Cardiac protein expression analysis

LV tissue samples from six HRP and six LRP rats were processed and the pooled samples obtained for each group were used for differential proteome analysis. In order to perform a 2DE comparative analysis, 3 large format 2DE gels were obtained for pooled samples of each group. An outline of the gel replicates is shown in the supplementary Figure S1. The spot pattern of the Coomassie blue staining was found to be comparable between groups.

The 2DE protein profiles obtained from HRP and LRP groups were highly reproducible regarding the total number of protein spots, their intensities and relative positions (correlation coefficient >0.95 for both groups). Image analysis of 2DE gels from HRP and LRP samples detected an average of 515 protein spots. No spot appeared exclusively on HRP or LRP gels. The factor analysis suggested differences in the protein profile between groups (data not shown).

**Figure 2.**
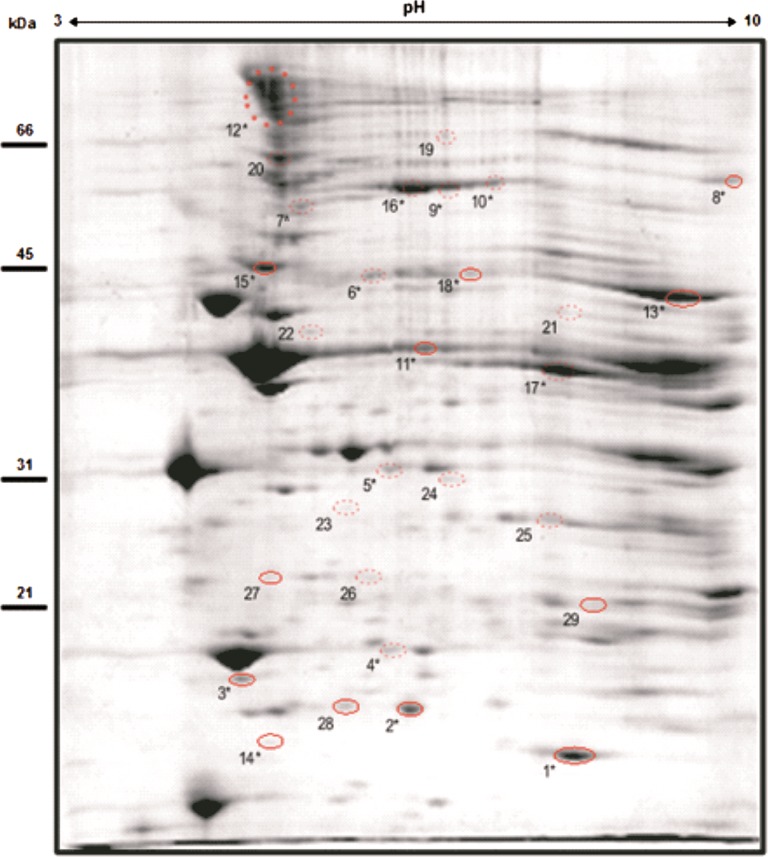
Representative 2-DE gel of the left ventricle protein extracts from high running performance (HRP) group. The spots numbered and surrounded are statistically different (P<0.05) between groups. The dotted circles show the spots with increased expression and complete circles show the spots with lower expression in the HRP group compared to the low running performance group. *Proteins identified by mass spectrometry (MS) and MS/MS.

The expression of 29 gel spots was significantly different between HRPs and LRPs. Among 29 candidate protein spots, 17 were upregulated and 12 were downregulated in the HRP group when compared to the LRP group. A representative 2DE gel from the HRP group is shown in Figure 2, in which numbers indicate significantly different spots between analyzed groups.

### Protein identification of differentially expressed protein spots

All 29 differentially expressed spots were excised from gels, digested and subjected to MALDI ToF/ToF. Protein identification by MS/MS was successfully obtained for 18 (62%) spots, in which at least 20% change in expression was observed between groups. The same gene product was identified in different spots, indicating a potential diversity of protein forms including post-translational modifications, mutations and/or isoforms. Thus, the 18 identified gel spots comprise 15 different gene products that were differentially expressed between HRP and LRP left ventricular tissue.

Identified proteins are indicated in Figure 2 by asterisks and listed in Table 2, along with the fold change between groups and categorization (biological processes). Information regarding the statistically significant fold change between groups and identified peptide sequences is shown in Supplementary Table 1.

**Table 2.** Data of differentially expressed proteins between high running performance (HRP) and low running performance (LRP) groups.

Spot	Protein name (ID)	Fold change^a^	Biological process^b^	Reported association
4	Myosin light chain -1 (P16409)	+1.6	Muscle contraction	Burniston et al. 13
7	Myosin heavy chain 6 (P02563)	+2.5	Muscle contraction	–
12	Myosin heavy chain 6 (P02563)	+1.7	Muscle contraction	–
17	Creatine kinase M-type (P00564)	+1.6	Muscle contraction	Momken et al. 22
11	α-actin (Q61272)	-2.7	Muscle contraction	Burniston et al. 11
1	α-crystallin B chain (P23928)	-1.3	Stress response	Burniston et al. 11
14	heat shock protein β-2 (O35878)	-1.5	Stress response	Noble 36
18	Aldehyde dehydrogenase 2 (P11884)	-1.9	Stress response	Burniston et al. 11
9	Serum albumin (P02770)	+2.1	Transport	Burniston et al. 13
10	Serum albumin (P02770)	+1.5	Transport	Burniston et al. 13
16	Serum albumin, isoform CRA_a	+1.2	Transport	–
5	Malate dehydrogenase (O88989)	+1.2	Carbohydrate metabolic process	–
15	Desmin (P48675)	-1.5	Intermediate filament organization	Burniston et al. 11
2	ATP synthase subunit d (P31399)	-1.3	ATP metabolic process	Boluyt et al. 23
13	ATP synthase subunit alpha (P15999)	-2.0	ATP synthesis, hydrogen ion transport	Burniston et al. 11;Bye et al. 7
8	Trifunctional enzyme subunit alpha (Q64428)	-3.0	Fatty acid metabolism	–
3	NADH dehydrogenase [ubiquinone] iron-sulfur protein 8 (O00217)	-1.3	Mitochondrial electron transport	Bye et al. 7
6	2-oxoglutarate dehydrogenase complex component E2 (Q01205)	+1.5	Tricarboxylic acid cycle	Boluyt et al. 23

a Ratio of the average normalized volume of spots with increased protein expression normalized by the average volume of spots with reduced protein expression. (+) Upregulation; (-) downregulation of spots in HRP group compared to LRP;

b Gene Ontology and PANTHER databases were used to define the biological processes.

The identified proteins fell into diverse functional categories and subcellular localization. Classification of these proteins using the subcellular localization database revealed that differentially expressed proteins comprised cytoplasmic, mitochondrial, cytoskeletal and myosin-complex related proteins. GO- and Panther-based biological process categorization revealed an involvement of these proteins in cell structure and muscle contraction, in the antioxidant metabolism and stress response, as well as in energy production (fatty acid oxidation, tricarboxylic acid cycle and oxidative phosphorylation). Notably, the HRP group showed upregulation of contractile proteins, such as myosin heavy chain-6, myosin light chain-1 and creatine kinase M-type, whereas LRP group showed upregulation of stress response related proteins, such as aldehyde dehydrogenase 2, heat shock protein β-2 and α-crystallin B chain, as well as desmin, which is the major protein component of the intermediate filaments in cardiac myocytes.

### Validation of proteomics data

**Figure 3.**
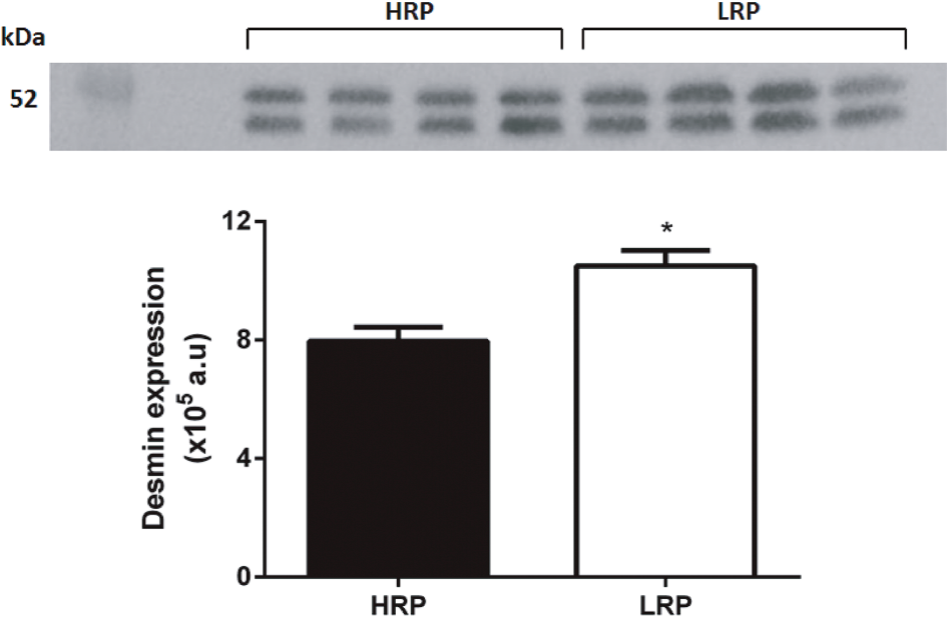
Desmin expression in left ventricle of low running performance (LRP) and high running performance (HRP) rats. Representative immunoblot (*top*) and densitometric analysis (*bottom*) showing changes in desmin protein levels. The membrane was stained with Ponceau S to document equitable protein sample loading. Data are reported means±SE (n=4); *P<0.01 (t-test).

Western blotting analysis of desmin was carried out in order to demonstrate its variation in individual rats, as well as to validate the proteomics data. The results from LV samples of four individual rats (from both HRP and LRP groups) demonstrated similar changes in desmin abundance, indicated by software differential image analysis of the 2D gels. Desmin was well detected as two bands in the immunoblotting results (Figure 3), and its upregulation in LRP phenotype was confirmed by densitometry analysis of the immunoblotting signals of the two bands, corroborating the results from our proteomic analysis.

## Discussion

This study revealed for the first time that there are differences in the LV proteome of non-selectively bred rats with distinct intrinsic exercise capacities (i.e., low running performance (LRP) versus high running performance (HRP)). Through 2DE/MS-MS analysis, we succeeded in identifying important proteins that are likely to be involved in the distinct intrinsic exercise capacity.

Both LRP and HRP animals had a frequency of ∼12% in the studied population, with HRPs showing a running capacity 3.5-fold greater than that of LRPs. Similar differences were observed at the 11th generation of HCR/LCR rats (6). No differences were observed, however, in body weight, heart weight and heart weight-to-body weight ratio between groups, which is in agreement with data describing rats with standard/high performance intrinsic aerobic exercise capacity (10).

The comparison between HRP and LRP LV proteomes identified changes in the expression of 29 protein spots, with 18 (62%) of these - accounting for 15 non-redundant proteins - being identified by MS/MS. The proteins identified are involved mainly in cell structure and muscle contraction, antioxidant metabolism/stress response, energy production and transport (Table 2). Some of our proteomic findings were consistent with existing knowledge, as some of these proteins have already been associated with intrinsic aerobic exercise capacity and cardiomyocyte contractile dysfunction in numerous independent studies (see references in Table 2).

Proteins involved in cell structure and muscle contraction - which could then affect myocardial contractility - represented a large proportion of the proteins differentially expressed between the distinct aerobic capacity phenotypes studied in this work. Two protein spots corresponding to myosin heavy chain 6 and one spot corresponding to myosin light chain-1 were markedly overexpressed in the HRP LV proteome (Table 2). Accordingly, several studies have described perturbations of the cytoskeleton in models of cardiac hypertrophy and failing myocardium, with changes in expression levels observed at both the transcriptional and translational levels (17,18).

A protein spot corresponding to creatine kinase (CK) M-type was also overexpressed in the HRP LV. CK M-type is a key intracellular enzyme that provides temporal and spatial energy buffering, playing a central role in the energy supply to several tissues. Its deficiency is a hallmark of cardiovascular diseases (19), suggesting that it is essential to myocardial energy homeostasis. Mice lacking CK isoforms (CK-/- mice) appeared to have nearly normal cardiac function under moderate workload (20), though the kinetics of caffeine-induced Ca^2+^ release and isoprenaline-responsive cardiac function were impaired in this model (21). These data suggest that CK is essential to the heart during an increase in energy demand, i.e., throughout exercise performance. In addition, increased CK may be one possible explanation for the improved transient Ca^2+^ found in endurance high-performance rats (10), for a proportion of this enzyme is known to be associated with sites of excitation-contraction coupling, providing energy for contractility and SR-Ca^2+^ reuptake (21,22).

Enzymes involved in energy production (malate dehydrogenase, 2-oxoglutarate dehydrogenase complex component E2) were also found to be significantly upregulated in HRPs. Similarly, dihydrolipoamide S-succinyl transferase was more abundant in the hearts of trained rats (23). The increase in expression of proteins involved in muscle structure/contraction and energy production observed in the LVs of the HRP rats may be important determinants for the high aerobic capacity of this inborn phenotype, by conferring improved cardiac function.

Alternately, we observed increased expression of three protein subunits from respiratory chain complexes I and V in LRP rats (NADH dehydrogenase ubiquinone iron-sulphur protein 8 and the ATP synthase d and alpha subunits). An increase in mRNA and protein levels of ATP synthase (mitochondrial F1 complex) and subunits from four of the respiratory complexes has been observed in LCRs and in a mouse model of hypertrophic cardiomyopathy (7,24). It has been suggested that the increase of ATP synthase subunits is a compensatory mechanism used to increase energy production (7).

Three protein spots corresponding to serum albumin were downregulated in the LRP group, which could be related to a shift in energy source from fatty acid β-oxidation to the metabolism of carbohydrates (7,11). An alternative explanation for the higher serum albumin in HRPs could be a greater vascular density, which would in turn lead to greater blood volume retention in the HRP versus LRP heart samples. Nonetheless, with the exception of the alpha subunit of the trifunctional enzyme, we have not identified other enzymes from the β-oxidation or glycolytic pathways. The lower level of this enzyme - three times less expressed in HRPs - is inconsistent with metabolic substrate for the energy supply (7,11). Modulation of short-chain specific acyl-CoA dehydrogenase was observed as multiple spots in HCR/LCR lines (11). The abundance of acid spots was two-fold greater in HCRs, whereas the more basic species were two-fold more abundant in LCR lines (11), which is consistent with our findings concerning the upregulation of the basic form of the trifunctional enzyme in LRPs.

In the present study, we observed that desmin and α-actin levels were also greater in the LRP group. Western blotting analysis of desmin - carried out to demonstrate individual variation as well as to add confidence to data on differential expression observed in 2D analysis from pooled samples - confirmed the upregulation of this protein in LRP phenotype, as similar changes in protein abundance were observed in biological replicates from individual animals. Curiously, the anti-desmin antibody employed in our western blot experiments detected two bands, which were also observed in immunoblotting profile studies previously reported (25), in which the lower band detected is thought to be a product of proteolytic cleavage of desmin.

Desmin is the main intermediate filament protein expressed in cardiac, skeletal and smooth muscle. It forms a continuous cytoskeletal network through its interaction with other proteins, ensuring that the contractile apparatus and other structural elements of the cell remain tuned, thus providing maintenance of cellular integrity force transmission, mechanic and chemical signaling (26). The abnormal accumulation of desmin may disturb the function of myofibrils, leading to unusual sarcolemmal tension, atypical distribution of organelles and the impairment of intra- and intercellular communication. Increases in its expression level have also been observed in hearts from LCRs (11) and in a mouse model of hypertrophic cardiomyopathy (HCM) (24). It was suggested that the overexpression of desmin in HCM mice is a compensatory or adaptive response to the loss of cell integrity in an attempt to reinforce the contractile units of the myocyte, thereby enhancing contractility (24). It has been suggested that desmin may act as an intracellular marker in heart failure (27). Recently, a study using modified mouse models found increased levels of desmin and actinin mainly in the myofibrils at the Z-disks of hearts with impaired diastolic function but not hearts with weakened systolic function (28).

Finally, we detected an increase in the expression of proteins associated with stress response in LCR rats, namely: aldehyde dehydrogenase 2 (ALDH2), α-crystallin B chain and heat shock protein (HSP) β-2. Alpha B-crystallin - a chaperone for desmin and cytoplasmic actin - is a small HSP that takes part in the maintenance of cytoskeletal integrity (29). The phosphorylation of α B-crystallin is associated with protection against a variety of different environmental stresses (30). In this instance, they are reduced to oligomeric size and translocated to selected myofibrillar proteins as well as to the cell membrane, where they are prone to protect the cytoskeleton (31). However, it is likely that α B-crystallin interacts with desmin after translocation, resulting in a cooperative protective role maintaining the cellular microstructure (32,33).

ALDH2 is best known as a detoxicant enzyme of ethanol metabolism. In addition, this enzyme eliminates other aromatic and aliphatic aldehydes, which are produced during lipid peroxidation (34,35).

HSPs are generally pro-survival molecules induced by a variety of stress agents, leading to protection effects. Events resulting in protein unfolding or denaturation, such as elevated temperatures, increased oxidative stress or metabolic disturbances can activate HSP response (36). Generally, exercise promotes an increased expression of the major HSPs (37).

The greater level of proteins involved in redox homeostasis in the LVs of LRP rats observed in this study may be due to mitochondrial dysfunction, with decreased energy production in these animals. Furthermore, a large body of research has highlighted the role of ALDH2 in cardioprotection by combating oxidative stress through a reduction of the cellular aldehydic load (reviewed in (38)). In addition, decreases in ATP levels have been previously found to elicit the induction of HSPs by DNA binding and activation of the transcriptional factor HSF1 (39).

These proteins are also capable of providing myocardial protection (37), acting in situations of extreme stress in order to prevent damage to cellular structures and tissue injury (40). Recently, an overexpression of antioxidant proteins and the presence of small HSPs in rats submitted to high-intensity resistance training have been reported (40).

Some of the limitations of the 2-DE proteomic/Coomassie approaches employed here are i) low protein resolution in the basic area of the gel, ii) protein extraction method not efficient for membrane proteins; iii) identification of only 62% of protein spots with differences in intensity. Nevertheless, the present study has shown with a great deal of certainty that there are several differences between HRP and LRP LV proteomes in non-selectively bred rats selected by aerobic capacity. Additionally, some cardiac proteins that increase with exercise training might be identified in animals born with high aerobic capacity, while proteins considered risk factors for cardiovascular disease are expressed in higher amounts in LRP animals. These data strongly corroborate the premise that inborn low aerobic capacity is likely to contribute towards cardiovascular disease and mortality.

## Supplementary material

Click here to view [pdf].
